# Deterioration of hematopoietic autophagy is linked to osteoporosis

**DOI:** 10.1111/acel.13114

**Published:** 2020-03-25

**Authors:** Ye Yuan, Yixuan Fang, Lingjiang Zhu, Yue Gu, Lei Li, Jiawei Qian, Ruijin Zhao, Peng Zhang, Jian Li, Hui Zhang, Na Yuan, Suping Zhang, Quanhong Ma, Jianrong Wang, Youjia Xu

**Affiliations:** ^1^ Department of Orthopaedics the Second Affiliated Hospital of Soochow University Suzhou China; ^2^ Osteoporosis Institute of Soochow University Suzhou China; ^3^ Research Center for Non‐medical Healthcare of Soochow University & Beijing Yaozhongtang Cyrus Tang Medical Institute Soochow University School of Medicine Suzhou China; ^4^ Hematology Center Cyrus Tang Medical Institute Soochow University School of Medicine Suzhou China; ^5^ Collaborative Innovation Center of Hematology Jiangsu Institute of Hematology Institute of Blood and Marrow Transplantation Institute of Neuroscience Key Laboratory of Stem Cells and Biomedical Materials of Jiangsu Province and Chinese Ministry of Science and Technology State Key Laboratory of Radiation Medicine and Radioprotection Soochow University School of Medicine Suzhou China

**Keywords:** autophagy, hematopoietic system, osteoporosis

## Abstract

Hematopoietic disorders are known to increase the risk of complications such as osteoporosis. However, a direct link between hematopoietic cellular disorders and osteoporosis has been elusive. Here, we demonstrate that the deterioration of hematopoietic autophagy is coupled with osteoporosis in humans. With a conditional mouse model in which autophagy in the hematopoietic system is disrupted by deletion of the Atg7 gene, we show that incapacitating hematopoietic autophagy causes bone loss and perturbs osteocyte homeostasis. Induction of osteoporosis, either by ovariectomy, which blocks estrogen secretion, or by injection of ferric ammonium citrate to induce iron overload, causes dysfunction in the hematopoietic stem and progenitor cells (HSPCs) similar to that found in autophagy‐defective mice. Transcriptomic analysis of HSPCs suggests promotion of iron activity and inhibition of osteocyte differentiation and calcium metabolism by hematopoietic autophagy defect, while proteomic profiling of bone tissue proteins indicates disturbance of the extracellular matrix pathway that includes collagen family members. Finally, screening for expression of selected genes and an immunohistological assay identifies severe impairments in H vessels in the bone tissue, which results in disconnection of osteocytes from hematopoietic cells in the autophagy‐defective mice. We therefore propose that hematopoietic autophagy is required for the integrity of H vessels that bridge blood and bone cells and that its deterioration leads to osteoporosis.

## Graphical abstract

Hematopoietic autophagy reduces osteoporosis by maintaining the integrity of H vessels that bridge blood and bone cells. It is therefore important to examine and improve hematopoietic autophagy capacity when considering clinical therapeutic interventions for osteoporosis.

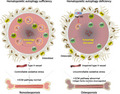

## 1 INTRODUCTION

Osteoporosis is an age‐related disease characterized by cumulative changes in bone metabolism that suppress bone formation and promote bone resorption (Rachner, Khosla, & Hofbauer, 2011; Reginster & Burlet, 2006). Together, these changes contribute to disordered bone homeostasis (Infante & Rodriguez, 2018; Wei & Sun, 2018). Bone homeostasis relies on a dynamic balance between osteogenesis by osteoblasts and osteoclastogenesis by osteoclasts (Cummings & Melton, 2002). It is well established that hematopoiesis, the production of functional blood cells, depends on both hematopoietic cells and nonhematopoietic cells in the bone marrow (Dorshkind, 1990). Nonhematopoietic stromal cells, including osteoblasts and osteoprogenitor cells, promote the maintenance of hematopoietic stem cells (HSCs) (Bowers et al., 2015; Morrison & Scadden, 2014). However, hematopoietic disorders can lead to bone deformities, and increasing long‐term survival prospects of patients with hematopoietic diseases, such as hematopoietic malignancies and β‐thalassemia, allows more complications of these diseases, such as osteoporosis, to develop (De Sanctis et al., 2018). The incidence of osteoporosis in allogeneic HSC transplantation recipients is about 50% (McClune & Majhail, 2013), and HSC transplantation increases the incidence of bone fractures (Pundole, Barbo, Lin, Champlin, & Lu, 2015). Recent clinical evidence also suggests that anemia due to aging is correlated with bone loss and fragility (Valderrabano et al., 2017; Valderrabano & Wu, 2019). All of this suggests a close connection between hematopoietic function and bone homeostasis, but direct links bridging the two in cellular function have yet to be found.

Autophagy is a genetically conserved mechanism for cellular homeostasis. There are three forms of autophagy: macroautophagy, microautophagy, and chaperone‐mediated autophagy. In microautophagy and chaperone‐mediated autophagy, small portions of cytosol components are engulfed or receive chaperone‐associated cargoes, respectively, for direct degradation in the lysosome. In contrast, macroautophagy is the major form of autophagy, and is therefore often termed autophagy. Macroautophagy is responsible for the turnover of organelles and portions of cytosol sequestered in a double‐membrane‐bound vesicle, the autophagosome, which slides along cytoskeletal structures and fuses with lysosomes, forming a single large and membrane‐surrounded vesicle called the autophagolysosome, where both the membrane and contents are degraded by lytic enzymes for recycling (Klionsky, 2005; Xie & Klionsky, 2007). By removing unnecessary cytoplasmic constituents, such as surplus mitochondria, endoplasmic reticula, peroxidases, and misfolded proteins, which are degraded by lysosomes via a lysosome‐degradation pathway (Mizushima, Levine, Cuervo, & Klionsky, 2008), autophagy prevents various cellular oxidative stresses that are major causes of aging.

The autophagy pathway includes a group of essential proteins, for example, ATG7, which helps in autophagosome formation (Suzuki & Ohsumi, 2007). With osteoblast‐specific Atg7‐conditional‐knockout mice, autophagy has been found to play a pivotal role in the maintenance of bone homeostasis, which involves elevated secretion of TNFSF11/RANKL (tumor necrosis factor superfamily, member 11) and decreased expression of TNFRSF11B/OPG (tumor necrosis factor receptor superfamily, member 11b) (Piemontese et al., 2016), as well as increased endoplasmic reticulum stress (Li et al., 2017). Insufficient autophagy in osteoporosis is associated with decreased SOD2 (Ezzat, Louka, Zakaria, Nagaty, & Metwaly, 2018), which is a strong anti‐aging enzyme. Autophagy is essential in maintaining the function of stem cells (Revuelta & Matheu, 2017) and in the hematopoietic system (Cao, Zhang, Cai, et al., 2015; Cao, Zhang, Yuan, et al., 2015; Gomez‐Puerto et al., 2016; Ho et al., 2017; Mortensen et al., 2011).

In this study, we found that hematopoietic deterioration is coupled with decreased bone mineral density in an aging human population. In addition, macroautophagy capacity in the hematopoietic system was significantly reduced in osteoporotic patients. With a genetically modified mouse model, we demonstrated that hematopoietic autophagy deterioration leads to osteoporosis.

## 2 RESULTS

### 2.1 Hematopoietic autophagy is deteriorated in osteoporotic patients

**Figure 1:**
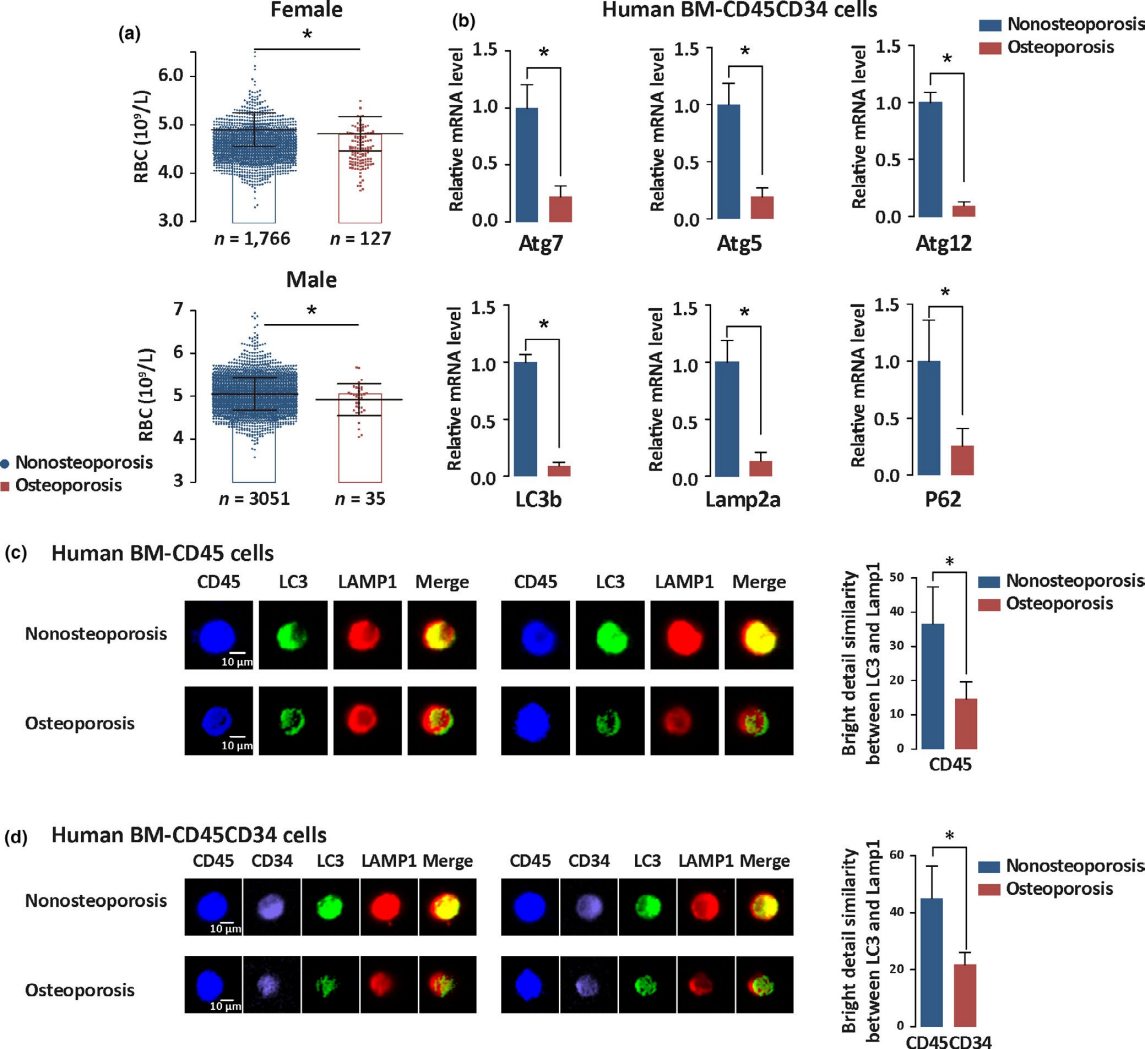
Bone marrow hematopoietic autophagy is decreased in osteoporotic patients. (a) Statistical analysis of bone mineral density (BMD) and red blood cell (RBC) count in human osteoporotic and healthy populations. Female nonosteoporosis individuals, *n* = 1,766; female osteoporosis individuals, *n* = 127; male nonosteoporosis individuals, *n* = 3,051; and male osteoporosis individuals, *n* = 35. BMD was tested by dual‐energy X‐ray absorptiometry. RBC count was examined by routine hematologic method.*p < .05. (b) Expression of autophagy‐essential genes in the bone marrow hematopoietic and stem cells of osteoporotic patients and healthy individuals. Osteoporosis was identified by BMD using dual‐energy X‐ray absorptiometry. BMD *T* value ≤−2.5 is a criterion for osteoporosis. Human femoral bone marrows were collected in total hip replacement or total knee replacement surgery. Bone marrow hematopoietic stem and progenitor cells were isolated with magnetic‐activated cell sorting (MACS) against CD45 and CD34 antibodies after Ficoll‐gradient separation. mRNA levels of Atg7, Atg5, Atg12, LC3b, Lamp2a, and P62 genes were detected by real‐time quantitative PCR. Data = means ±*SD*s. *p < .05. *n* = 30/group. (c, d) Detection of autolysosome formation in healthy and osteoporotic populations. Autolysosome formation was measured by image flow cytometry for double staining of LC3 and lysosome with bone marrow CD45 or CD45CD34 cells prepared by fluorescence‐activated cell sorting (FACS). Autolysosome formation was represented by the colocalization of LC3 and lysosomal marker LAMP1. Left panel, representative flow image; right, results of flow image analysis. Data = means ± *SD*s. *p < .05. *n* = 30/group

To explore the cellular connection between osteoporosis and the decline in hematopoietic function, we first compared red blood cell (RBC) counts between osteoporotic patients and healthy individuals. Osteoporosis was defined based on decreases in bone mineral density (BMD), per WHO standards (BMD *T* value ≤−2.5), measured by dual‐energy X‐ray absorptiometry (DXA), and the RBC counts were obtained from routine blood tests for both osteoporotic patients and healthy individuals. RBC counts of 1,766 female nonosteoporosis, 127 female osteoporosis, 3,051 male nonosteoporosis, and 35 male osteoporosis individuals were analyzed. The results showed that RBC count was reduced in both male and female osteoporotic patients, with no obvious differences between the two sexes (Figure 1a).

To determine whether hematopoietic autophagy capacity differs between the healthy population and osteoporotic patients, participants were divided into a nonosteoporosis group and an osteoporosis group. We used the cell surface molecule CD45 to characterize hematopoietic cells, and CD45CD34 double‐cell surface molecules to characterize CD34‐enriched human HSCs by magnetic bead separation or fluorescence‐activated cell sorting (FACS). Human bone marrow samples were collected from hip replacement or knee replacement surgery. Patients were divided into nonosteoporosis (BMD *T* value ＞−2.5) or osteoporosis (BMD *T* value ≤−2.5) group. Expression of a group of autophagy‐essential genes (Atg) in the primary human HSCs was measured by quantitative PCR. Results showed that virtually all autophagy‐essential genes were downregulated in their transcription in the osteoporosis group compared to the nonosteoporosis group, both of which include both male and female individuals (Figure 1b). The reduction of autophagy gene expression and autophagic activity displayed a similar pattern in the mixed group of males and females to that in the single‐sex group (Figure S1), suggesting that the hematopoietic autophagy machinery is inhibited in osteoporotic patients, with minimal preference based on sex.

We next used imaging flow cytometry (ImageStream) to analyze functional macroautophagic capacity in human bone marrow hematopoietic cells and their stem cells. This capacity is represented by the colocalization of tubulin light chain 3 (LC3) and the lysosomal marker LAMP1 (Phadwal et al., 2012), a key step for macroautophagy mediated by ATG8 and ATG12 conjugation systems in which ATG7 plays a critical role (Xie & Klionsky, 2007). Consistent with the decreased expression of Atg genes, the colocalization of LC3 and lysosomes was reduced in osteoporotic bone marrow CD45 hematopoietic cells (Figure 1c) and bone marrow CD34‐enriched HSCs (Figure 1d). Taken together, these results indicate that bone marrow hematopoietic macroautophagy, hereafter referred as autophagy, is decreased in osteoporotic patients, suggesting that osteoporosis is highly associated with reduced autophagy in the hematopoietic system.

### 2.2 Induced bone loss is associated with hematopoietic abnormality in mice

**Figure 2:**
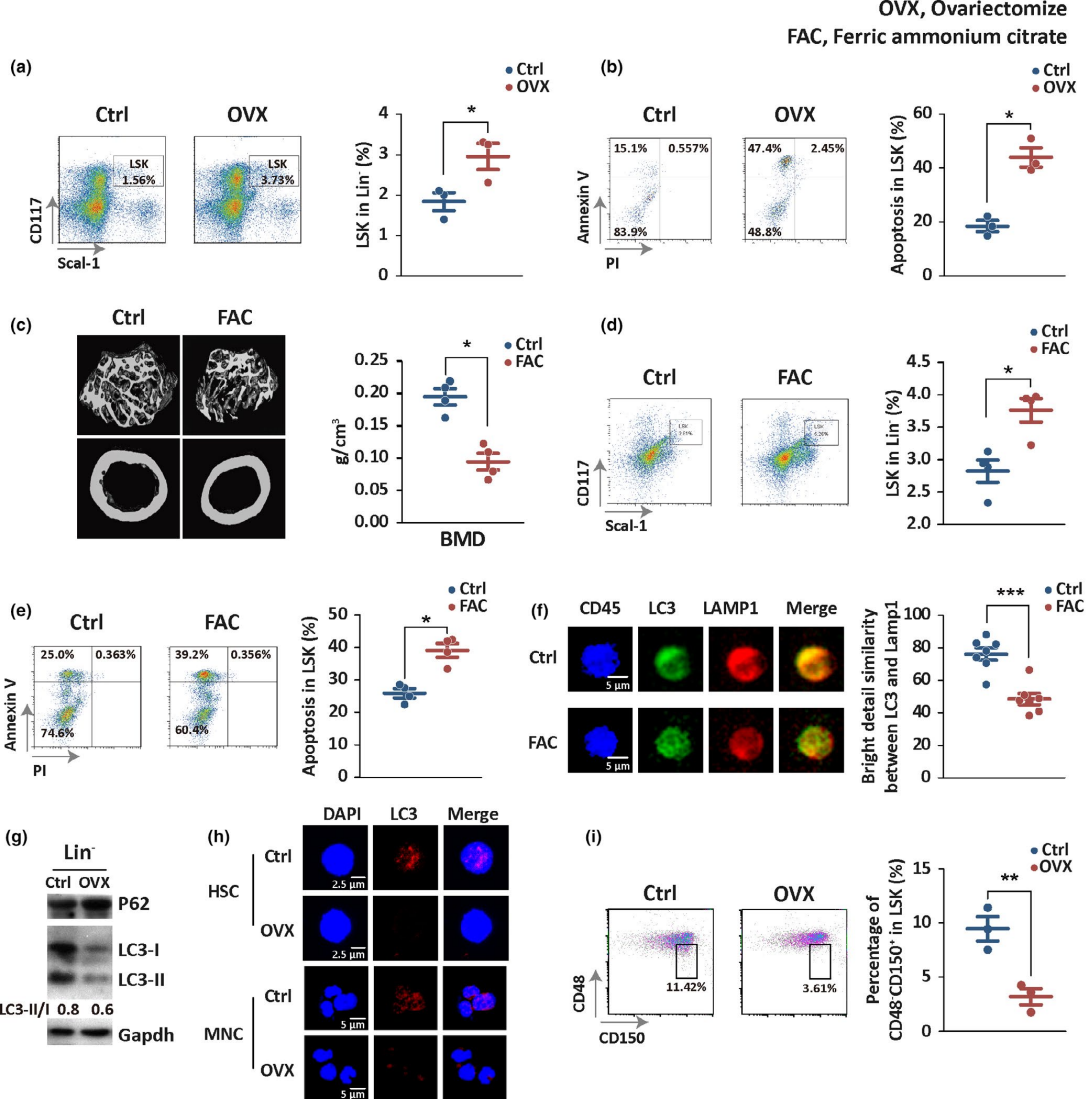
Induced osteoporosis resembles the hematopoietic autophagy defect‐caused phenotype in bone loss and hematopoietic abnormality. (a, d) HSPC proportion was detected for mice in three groups by flow cytometry: wild‐type (Ctrl), ovariectomy (OVX), and iron accumulation (FAC). Generation of both mouse models started at 8 weeks of age. The OVX group and its control were sacrificed at 14 weeks of age, while FAC group and its control were sacrificed at 16 weeks of age for analysis. Bone marrow was isolated from femur and tibia for analysis. Left panel, representative images showing percentage of Lin^‐^CD117^+^ Scal‐1^+^ in Lin^−^ cells; right panel, graph showing the significant difference between control and model groups. *p < .05. (b, e) Detection of apoptosis of HSPCs in the same three groups by flow cytometry. Left panel, representative images showing apoptosis in HSPCs; right panel, graph showing the significant difference between control and model groups. *p < .05. (c) The representative micro‐CT reconstructed three‐dimensional pictures of femur trabecular and cortical bones. Femora were collected from wild‐type and iron accumulation mice. (f) Functional colocalization of autophagosome marker LC3 and lysosome marker LAMP1 in the CD45‐positive hematopoietic cells sorted from the control and FAC mice, measured by image flow cytometry with fluorescent antibodies against LC3 or LAMP1. Left panel, representative images showing colocalization of LC3 and LAMP1; right panel, graph showing the significant difference between control and FAC mice. ***p < .001. *n* = 5. (g) P62 and LC3 protein expression in Lin^−^ cells of OVX mice. (h) Confocal microscopic detection of LC3 puncta by immunostaining of DAPI and LC3 in bone marrow hematopoietic stem cells (HSC) and mononuclear cells (MNC) of OVX mice. (i) Percentage of HSCs (CD48^−^ CD150^+^ LSK) in HPCs (LSK) was detected in OVX mice. Left panel, representative images showing percentage of CD48^−^ CD150^+^ in LSK compartment; right panel, graph showing the significant difference between control and OVX mice. **p < .005

In order to support the observation in patients that osteoporosis is associated with dysfunction of hematopoietic autophagy, we evaluated bone marrow hematopoietic function in ovariectomized (OVX) mice, a traditional animal model for postmenopausal osteoporosis (Calado et al., 2009; Rachner et al., 2011). Eight‐week‐old female mice were ovariectomized and sacrificed 6 weeks later. The control group was performed as sham surgery. The proportion of hematopoietic stem and progenitor cells (HSPCs) increased in OVX mice (Figure 2a), but the apoptosis of HSPCs also increased (Figure 2b). The phenotypes for HSPCs in OVX mice mimic those in the hematopoietic autophagy‐defective mice (Cao, Zhang, Cai, et al., 2015).

Iron accumulation in menopausal females often increases risk in osteoporosis, largely attributed to less loss of blood (Kim et al., 2012; Rachner et al., 2011; Tsay et al., 2010; Xiao et al., 2015; Yuan et al., 2019). To determine whether osteoporosis from iron overload is also associated with hematopoietic dysfunction at the HSPC level, we generated iron accumulation‐induced osteoporotic mice (Tsay et al., 2010). Eight‐week‐old male mice were treated with 0.1 g kg^−1^ week^−1^ of ferric ammonium citrate (FAC) for 8 weeks. The control group was treated with normal saline simultaneously. Micro‐CT for three‐dimensional reconstruction showed bone loss in the iron accumulation mice (Figure 2c), indicating a successful induction. Consistent with that in the OVX mouse model, the frequency of HSPCs increased (Figure 2d), but the apoptosis of the HSPCs also increased in the iron‐overloaded mice (Figure 2e). ImageStream measurement that quantified autophagy activity represented by functional colocalization of LC3 and LAMP1 suggested that the formation of autolysosomes was significantly reduced in the hematopoietic cells of the ferric ammonium citrate (FAC)‐treated mice (Figure 2f).

To test whether hematopoietic autophagy is defective in the osteoporotic mouse model as it does in osteoporotic patients, we isolated bone marrow Lin^‐^ cells and HSPCs from the OVX model and control mice, immediately followed by examination with Western blotting and confocal microscopy. In the OVX mouse model, P62, an autophagy‐specific substrate, was apparently accumulated, but LC3‐I expression and its conversion to LC3‐II were reduced (Figure 2g), suggesting a considerable autophagy defect in the hematopoietic cells of the OVX mice. Microscopic results further indicate that the bone marrow hematopoietic stem cells (HSCs) and mononuclear cells of the osteoporotic mouse model displayed a significant decrease in the formation of the LC3 puncta (Figure 2h), again suggesting a reduced basal autophagy activity in the hematopoietic system from the osteoporotic mice.

A pioneering study by Mortensen and colleagues indicates a decreased HSC frequency in HSPCs in hematopoietic autophagy‐defective mice (Mortensen et al., 2011). In our previous work, we examined HSC number and frequency within Lin‐negative cells and found that short‐term HSCs, the majority of HSCs (about 90% of total HSCs), were reduced in both number and frequency, whereas long‐term HSCs (about 10% of total HSCs) were increased in both number and frequency. Thus, autophagy defect leads to an overall decrease in frequency of HSCs (Cao, Zhang, Cai, et al., 2015). In the present study, we found that overall HSC frequency in HSPCs (LSK compartment) was also reduced in the OVX osteoporotic mice (Figure 2i**)**. These observations in mouse models again suggest a functional connection between hematopoietic autophagy defect and osteoporosis.

Taken together, these results indicate that osteoporosis models generated by either ovariectomy or iron overload have negative impacts on HSCs or HSPCs similar to those in osteoporosis patients with hematopoietic autophagy deterioration and in genetically modified mice with hematopoietic autophagy defect, suggesting that hematopoietic autophagy dysfunction and osteoporosis are linked.

### 2.3 Genetic disruption of autophagy in the hematopoietic system causes bone loss in mice

**Figure 3:**
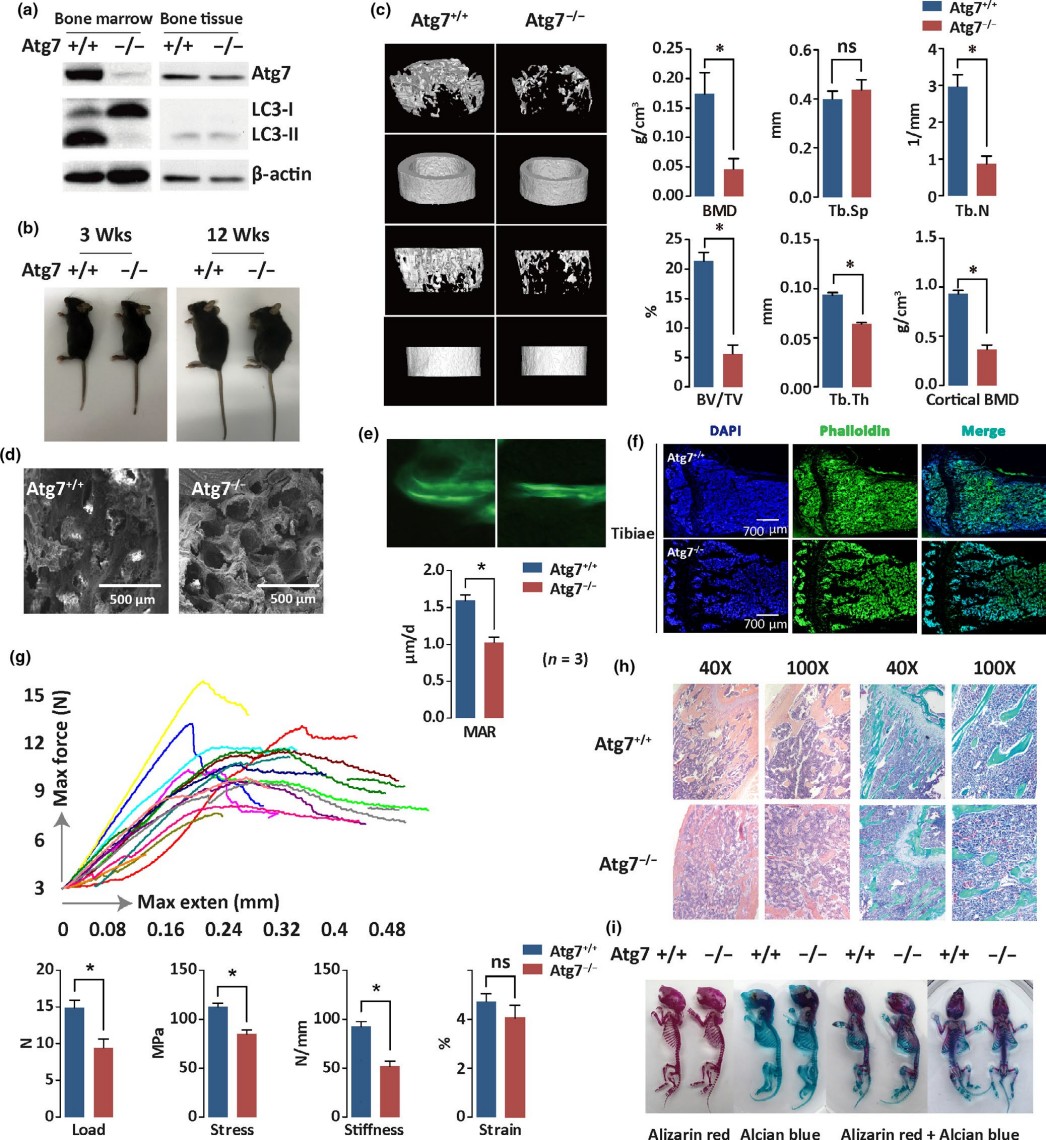
Hematopoietic autophagy defect caused severe bone loss. (a) Confirmation of autophagy disruption in the Atg7^−/−^ mice. Bone marrow and bone tissue from 10‐week‐old mice were collected for Western blotting analysis of ATG7 and LC3. β‐Actin was used as a loading control. (b) Representative images of wild‐type and Atg7‐deleted mice at ages of 3 or 12 weeks. *n* = 3. (c) Micro‐CT analysis. Femora were collected from 8‐week‐old mice. Left, representative reconstructed three‐dimensional pictures of femur trabecular and cortical bones. Right, micro‐CT quantification of the trabecular index of femur samples. Measurement of distal femur spatial structure parameters include bone mineral density (BMD), trabecular space (Tb.Sp), trabecular number (TB.N), bone tissue fraction (BV/TV), trabecular thickness (Tb.Th), and cortical thickness. *p < .05. *n* = 3. (d) Scanning electron microscopy (SEM) analysis of trabecular microstructure of femur. Femora were collected from 8‐week‐old mice. *n* = 3. (e) Representative calcein double‐labeling images with quantification of mineral apposition rate (MAR). *n* = 3. (f) Immunofluorescence of 8‐week‐old mouse tibia metaphysis frozen section stained with phalloidin antibody (green) and DAPI (blue). Fluorescently tagged phalloidin staining was performed to measure the density of osteocyte cell projection formed during the process of embedding into bone matrix. Nuclei were visualized by DAPI staining. *n* = 3. (g) Three‐point bending test for bone biomechanical properties. Femora were collected from 8‐week‐old mice and were measured for load, stress, stiffness and strain. Upper panel, representative image; lower panel, bar graph. *p < .05. *n* = 9. Femora were collected from 8‐week‐old mice. *p < .05. *n* = 6. (h) HE and Masson staining of tibia paraffin section. Tibiae were collected from 8‐week‐old mice for paraffin section and immunohistochemistry. *n* = 6. (i) Alizarin red/Alcian blue staining of the whole‐body skeleton from neonatal mice. Mice were sacrificed at postnatal day 3. Skeleton was stained after soft tissue was removed. *n* = 3

To explain the observation that hematopoietic autophagy is deficient in osteoporotic patients and that hematopoietic dysfunction occurs in induced osteoporotic mice, we used a conditional mouse model (Atg7^−/−^) in which Atg7 had been genetically deleted in the hematopoietic system for phenotypic analysis. Atg7 is an autophagy‐essential gene required for autophagy machinery that is essential in the hematopoietic system (Cao, Zhang, Yuan, et al., 2015; Mortensen et al., 2011). Deletion of Atg7 impaired autophagy, shown by the missing Atg7 band and the lack of lipidation of LC3‐I to LC3‐II in total bone marrow hematopoietic cells, which shows a very faint band for LC3‐II (Figure 3a, left). The Atg7 gene was, however, not deleted in the bone tissue of the mouse model, as detected by Western blotting that showed no change in Atg7 protein level between wild‐type (Atg7^+/+^) and mice with the Atg7 gene deleted (Atg7^−/−^) in bone tissue (Figure 3a, right). Further examination of the tibia cortical bone by immunofluorescence with the ATG7 antibody confirmed no Atg7 deletion in the bone tissue (Figure S2), thereby validating the mouse model to study the impact of defective hematopoietic autophagy on its skeletal system.

Apart from disabled hematopoietic stem cell capacity (Figure S3), deficient hematopoietic autophagy induced by genetic modification of the Atg7 gene caused obvious hunchback, suggesting spinal abnormality (Figure 3b). Micro‐CT scanning of the femur trabecular and cortical bones in the mouse revealed severe bone loss in the Atg7^−/−^ mice (Figure 3c, left). We used micro‐CT to analyze distal femur trabecular bone reconstruction of the three‐dimensional form and spatial structure parameter. By 8 weeks of age, animals lacking autophagy in the hematopoietic system had significantly reduced BMD, which was associated with a decrease in trabecular number (TB.N), bone tissue fraction (BV/TV), trabecular thickness (Tb.Th), and cortical thickness; no significant change was detectable in the trabecular space (Tb.Sp) (Figure 3c, right). Thus, the reconstruction of three‐dimensional forms again revealed severe bone loss and inhibition of mineralization in the hematopoietic autophagy‐defective mice.

Consistent with the above observation, a scanning electron microscopy (SEM) analysis indicated abnormal trabecular microstructure of the femur in the Atg7^−/−^ mice. Hematopoietic autophagy defect induced significant bone loss in trabecular bone areas and reduced bone density (Figure 3d); calcein double labeling, used for measuring bone mineralization (Shimada et al., 2018; Yue, Shen, & Morrison, 2016), revealed a reduced rate of trabecular bone formation in the Atg7^−/−^ mice (Figure 3e); immunofluorescence assay with phalloidin antibody revealed abnormal bone marrow microstructure in the metaphysis and trabecula in the Atg7^−/−^ mice (Figure 3f); and a three‐point bending test for bone biomechanical properties indicated deteriorated bone quality in the Atg7^−/−^ mice (Figure 3g). Finally, HE and Masson staining of the tibiae showed broken collagenous fiber and elastic fibers in the Atg7^−/−^ mice, confirming destroyed bone microstructure (Figure 3h). All together, these results demonstrate that hematopoietic autophagy deficiency causes reduced bone formation and severe bone loss.

To determine whether the bone loss was due to inhibition in the development of early bone, we used Alizarin red/Alcian blue staining to visualize the skeletal system of Atg7^+/+^ and Atg7^−/−^ mice sacrificed at postnatal day 3. Virtually, no distinguishable difference in the ossified region of bones was found between the wild‐type mice and the autophagy‐defective mice (Figure 3i). This result suggests that hematopoietic autophagy defect‐caused bone loss is not due to an embryonic developmental disorder in the skeletal system; rather, it is likely caused by a postnatal accumulative insult from a hematopoietic autophagy deficiency.

### 2.4 Hematopoietic autophagy deficiency impairs osteocyte homeostasis in mice

**Figure 4:**
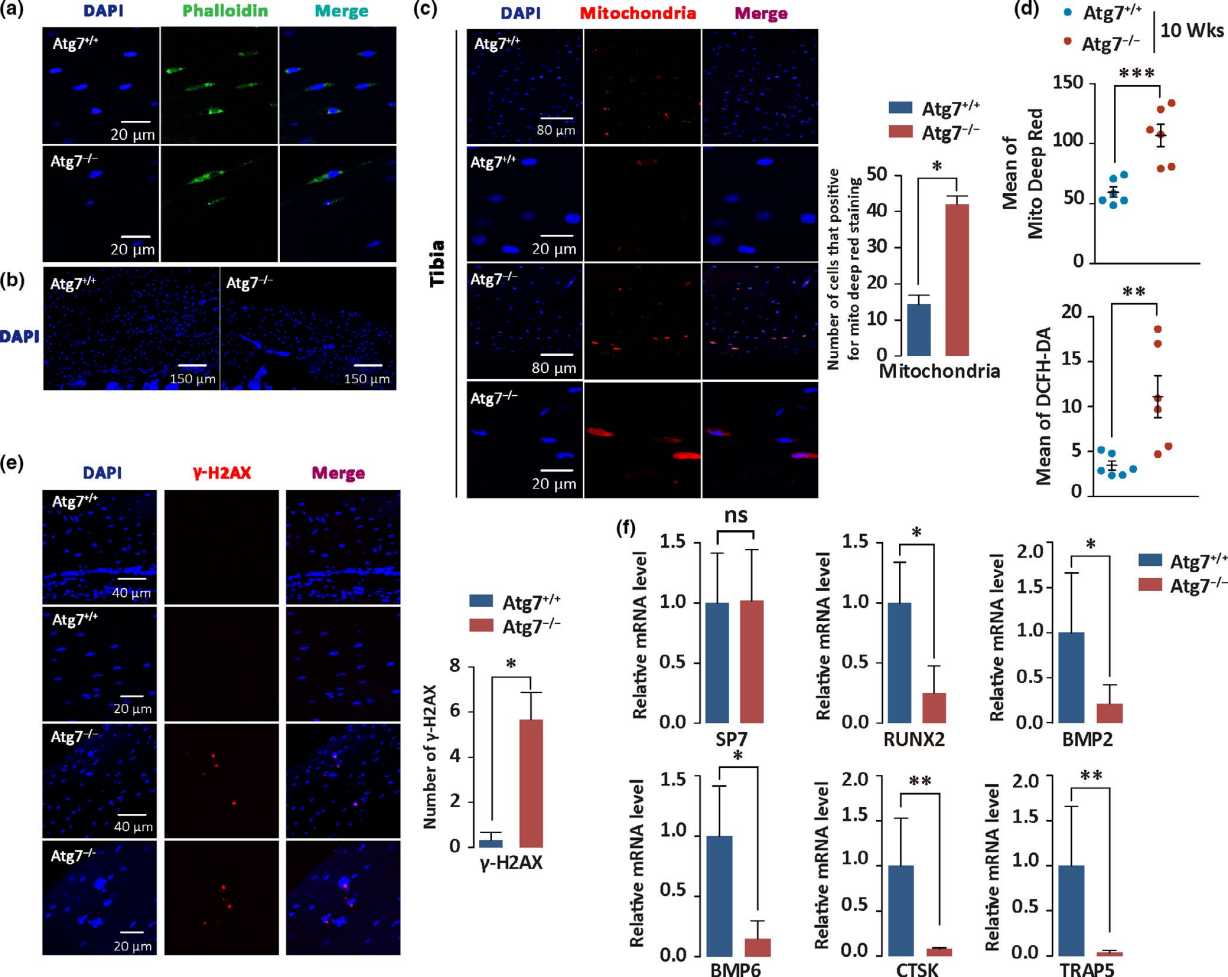
Hematopoietic autophagy defect disturbs osteocyte homeostasis. (a) Immunofluorescence of 8‐week‐old mouse tibia frozen section stained with phalloidin antibody (green) and DAPI (blue). Fluorescently tagged phalloidin staining was performed to measure the density of osteocyte cell projections formed during the process of embedding into bone matrix. Nuclei were visualized by DAPI staining. *n* = 3. (b) Immunofluorescence of 8‐week‐old mouse tibia cortical bone frozen section stained with DAPI (blue), reflecting the density of osteocytes per bone area. *n* = 3. (c) Immunofluorescence of 8‐week‐old mouse tibia cortical bone frozen section stained with MitoTracker Deep Red (red) and DAPI (blue). Right, quantitative data for mitochondrial level measured from microscopic images. *n* = 3. (d) Flow cytometric detection of mitochondrial mass and ROS levels. Bone cells were prepared from femurs of 8‐week‐old mice by digesting with trypsin and collagenase I. Mitochondrial mass and ROS levels were measured with MitoTracker Deep Red and DCFH‐DA on a flow cytometer, respectively. For mitochondria staining, red represents mitochondria and blue represents DAPI. Merged cells in mitochondria staining were quantified. **p < .005, ***p < .001. *n* = 6. (e) Immunofluorescence of 8‐week‐old mouse tibia cortical bone frozen section stained with γ‐H2AX antibody (red) and DAPI (blue). Merged cells in γ‐H2AX staining were quantified (right). *n* = 3. (f) Measurement of expression levels for several genes critical in regulation of osteocyte homeostasis in bone tissue. Femora and tibiae were collected from 8‐week‐old control and Atg7‐deleted mice for total RNA extraction. The gene encoding proteins examined by real‐time quantitative PCR include SP7, RUNX2, BMP2, BMP6, CTSK, and TRAP5 in bone tissue. The primers used for amplification of these genes are given in the methods (Table S1). SP7 (a bone specific transcription factor) is essential for osteoblast differentiation and bone formation; RUNX2 (runt related transcription factor 2) is essential for osteoblast differentiation and skeletal morphogenesis; BMP2 (bone morphogenetic protein 2) and BMP6 (bone morphogenetic protein 6) both regulate bone development; BCTSK (cathepsin K) and TRAP5 (tartrate‐resistant acid phosphatase 5) are required in bone remodeling and resorption. mRNA levels are normalized to GAPDH. *p < .05, **p < .005. *n* = 3

Osteocyte homeostasis is the basis for bone tissue homeostasis. To determine the impact of hematopoietic autophagy on bone homeostasis in adult mice, tibia was collected for frozen section to examine bone histomorphology by fluorescent phalloidin that stains F‐actin, and DAPI that stains nuclei. The results showed reduced density of osteocyte cell projections in the Atg7^−/−^ mice, reflected by the size and number of osteocytes, revealing an attenuated formation or maintenance of the osteocyte network (Figure 4a). DAPI staining also showed decreased osteocytes in the cortical bone of the Atg7^−/−^ mice (Figure 4b).

Cellular reactive oxygen species (ROS), largely produced in mitochondria, are known to regulate signaling pathways that govern osteocyte differentiation (Atashi, Modarressi, & Pepper, 2015), but surplus ROS disturb cellular homeostasis (Li et al., 2017; Sheweita & Khoshhal, 2007). We therefore examined tibia osteocyte mitochondrial level by immunofluorescence staining with MitoTracker Deep Red. The results showed higher mitochondrial mass, representing increased oxidative stress in the cortical bone osteocytes of the autophagy‐defective mice (Figure 4c). We also measured mitochondrial mass and oxidative levels in the femur bone tissue of mice by flow cytometry. Results showed an increased mitochondrial mass and an elevated ROS level in the bone tissues of the hematopoietic autophagy‐defective mice (Figure 4d), suggesting that the bone loss may be due to oxidative insults in their bone tissue.

We then used immunofluorescence to stain γ‐H2AX, which is a typical hallmark for DNA damage. Increased positive staining was seen in tibia osteocytes of the autophagy‐defective mice, suggesting that osteocytes experienced increased DNA damage that may have initially stemmed from defective hematopoietic autophagy (Figure 4e). Next, we used quantitative PCR to measure the expression of key factors regulating osteocyte homeostasis, including SP7, RUNX2, BMP2, BMP6, CTSK, and TRAP5. The results revealed significant reduction of these factors in the femora and tibiae in the Atg7^−/−^ mice (Figure 4f), suggesting that autophagy deficiency in the hematopoietic system disturbs multiple pathways that govern osteocyte homeostasis.

### 2.5 Transcriptomic profiling of HSPCs suggests inhibition in calcium metabolism and osteocyte differentiation and elevation in iron activity from hematopoietic autophagy defect in mice

To explore the basis of functional connection between hematopoietic autophagy and osteocyte homeostasis, we performed RNA sequencing of the HSPCs of wild‐type and Atg7‐deleted mice. HSPCs sorted with LSK markers were collected from 10‐week‐old mice femur and tibia bone marrow for RNA sequencing. Transcriptomic profiling showed that disruption of the Atg7 gene in the hematopoietic system led to downregulation of osteocyte differentiation and calcium metabolism since an array of genes responsible for positive regulation of osteoclast differentiation and osteoblast differentiation and calcium signaling and transport were downregulated (Figure S4A). This result suggests that intact autophagy in hematopoietic cells is essential for osteocyte homeostasis, which is consistent with the results of phenotypic analysis in the Atg7‐deleted mice (Figures 2,3 and 4). However, this observation is in contrast to existing understanding in osteogenesis, represented by a recent study, showing that loss of the hematopoietic stem cell factor GATA2 in the mesenchymal stem cells impairs trabecularization and mechanical strength of bone (Tolkachov et al., 2018). Surprisingly, deletion of Atg7 in the hematopoietic system enhanced iron activity in the HSPCs (Figure S4), which is often related to iron overload‐induced osteoporosis.

### 2.6 Proteomic profiling of bone tissue reveals extensive inhibition in multiple pathways regulating bone homeostasis in the hematopoietic autophagy‐defective mice

To confirm the observations from RNA sequencing of the HSPCs, we performed proteomic analysis of the bone tissues of the mice. Specifically, we used the tandem mass tags approach to quantify the proteome of femora from wild‐type and hematopoietic autophagy‐defective mice. The repeatability of proteomic analysis was consistent across samples (Figure S5). In total, 4,221 protein groups were identified, among which 3,602 proteins were quantified. The fold‐change cutoff was set for proteins with quantitative ratios above 1.3 or below 0.77. Among quantifiable proteins, 218 proteins were upregulated, and 192 proteins were downregulated. Biological process analysis revealed a significant reduction in the proteins involved in collagen fibril organization, endochondral bone morphogenesis, bone morphogenesis, and skeletal system morphogenesis in the bone tissue of the hematopoietic autophagy‐defective mice (Figure S6). Functional annotation displayed downregulation of numerous proteins involved in bone homeostasis (Figure S7).

**Figure 5:**
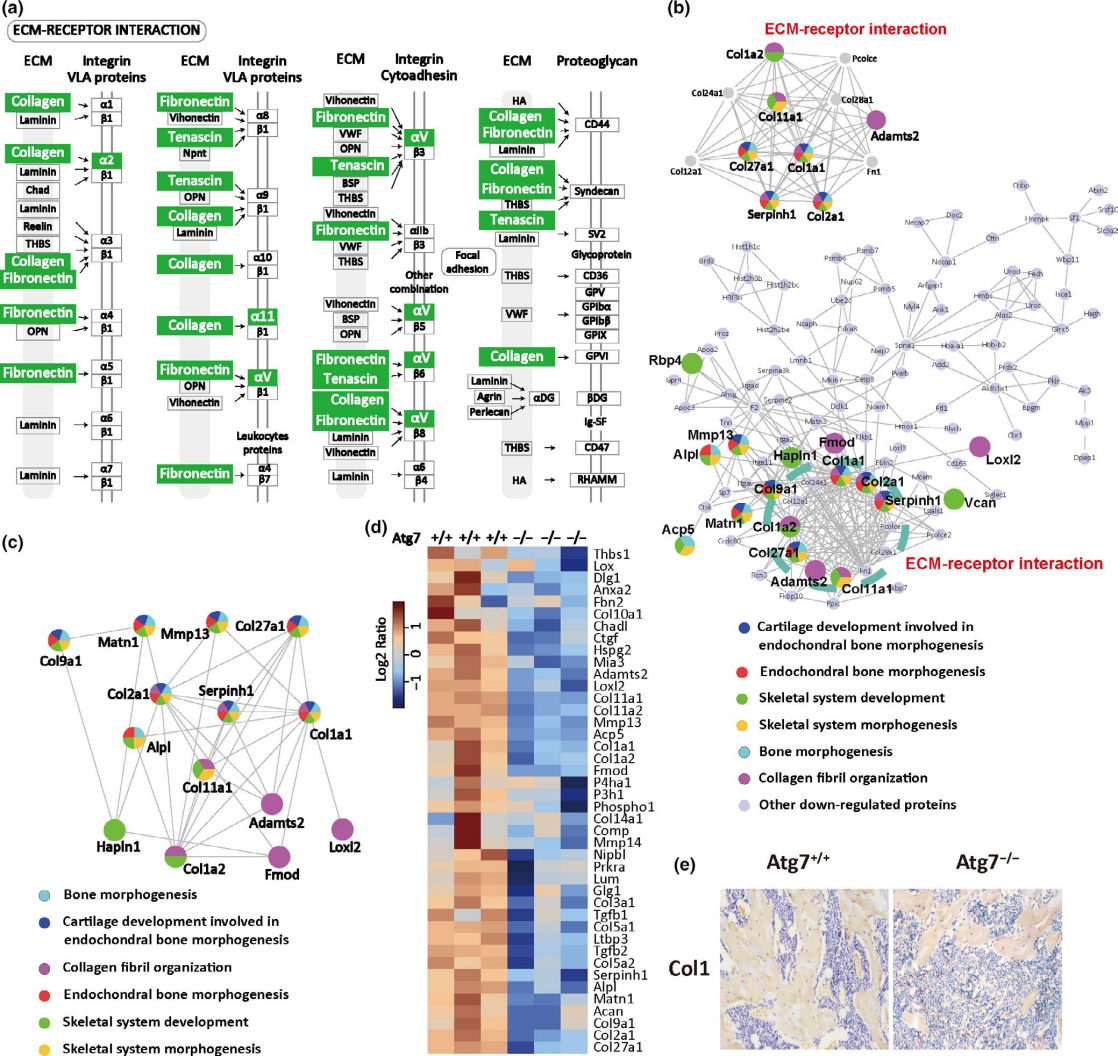
Proteomic profiling of tibia bone of hematopoietic autophagy‐defective mice. (a) KEGG analysis of extracellular matrix (ECM) pathway. (b) STRING analysis of interaction network for downregulated proteins in ECM function. (c) STRING analysis of interaction network for downregulated collagen family members. (d) Heatmap analysis of the altered expression levels for proteins in the ECM pathway. (e) Collagen 1 staining of tibia section. The tibiae were collected from 8‐week‐old mice for immunohistochemistry. Collagen 1 was stained golden yellow

The extracellular matrix (ECM) is a predominant pathway regulating bone homeostasis (Nesbitt & Horton, 1997). KEGG analysis on the autophagy deficiency mouse model revealed clear inhibition of the ECM pathway, in which many proteins (highlighted in green) were downregulated (Figure 5a). STRING analysis of the interaction network again revealed many downregulated proteins in the ECM function in the bone tissue of the Atg7^−/−^ mice (Figure 5b,c). A protein annotation heatmap revealed a significant decrease in an array of proteins that regulate bone homeostasis, and surprisingly, almost all of the collagen family members in the array were downregulated in the bone tissue of the Atg7^−/−^ mice (Figure 5d). To validate the bioinformatic information, we used immunohistochemistry to examine the expression of Collagen 1 in the tibia section of the mice, and the reduction in its expression was confirmed in the Atg7^−/−^ mice (Figure 5e). Collagen family members in the ECM pathways play critical roles in osteogenesis and osteoclast bone resorption (Nesbitt & Horton, 1997). For example, Collagen 1 is known to be important in aging and osteoporosis (Kawana, Takahashi, Hoshino, & Kushida, 2002). Thus, the proteomic profiling suggests that the ECM–receptor interaction was disrupted due to inhibited collagen expression in the hematopoietic autophagy‐defective mice.

### 2.7 Hematopoietic autophagy defect causes disruption of H vessels in the bone tissue

**Figure 6:**
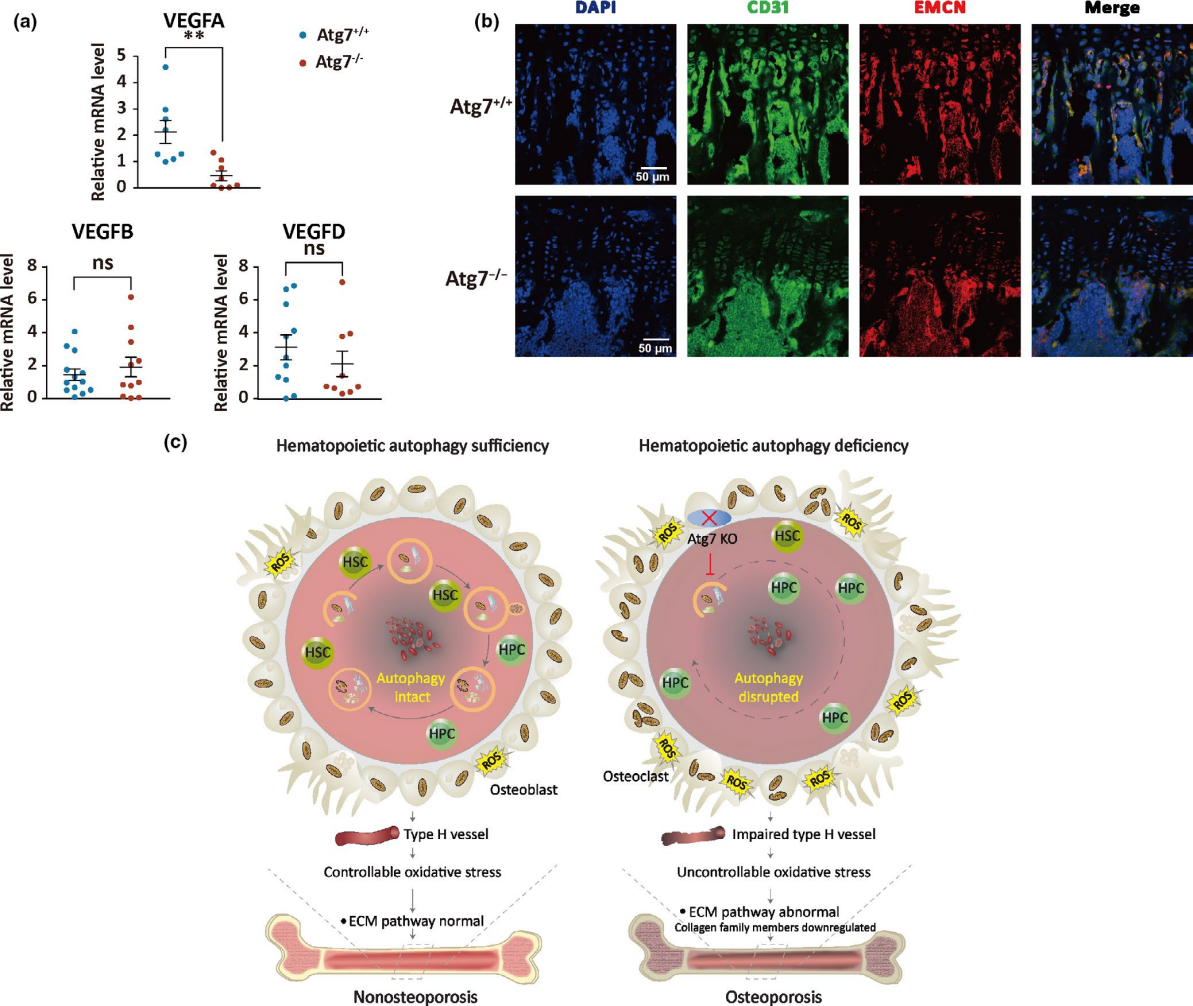
Examination of angiogenesis in bone tissues of the autophagy‐defective mice. (a) Expression of Vegf gene family members in Atg7^+/+^ and Atg7^−/−^ bone tissue. The mRNA levels for analysis are normalized to the mRNA encoding GAPDH. **p < .005. (b) Identification of type H vessels. Type H vessels are a major connection between blood cells and bone formation. 8‐week‐old mouse tibia metaphysis frozen section was stained with CD31 antibody (green), EMCN antibody (red), and DAPI (blue). CD31 and EMCN merged colors represent type H vessels. Shown are the representative images by immunofluorescence from WT and Atg7‐deleted mice. *n* = 3. (c) A cartoon illustrating the functional connection between hematopoietic autophagy and prevention of osteoporosis. Sufficient hematopoietic autophagy maintains homeostasis in both the hematopoietic system and bone tissue. Deficient hematopoietic autophagy leads to not only abnormal hematopoiesis, but also concurrently impaired type H vessels, elevated mitochondrial mass, and oxidative stress that together disturb the ECM pathway. Ultimately, it leads to accelerated osteoporosis

Proteomic profiling revealed connective information in disordered expression of the proteins pertinent to bone metabolism. Nevertheless, it is unable to detect the signals of proteins with low expression due to technical incapacity in the current study. Since H vessels are a major connection between blood cells and osteocytes, we hypothesized that hematopoietic autophagy defect would compromise the integrity of the vessels. To study this, we examined key genes regulating angiogenesis, with a focus on the family members of vascular endothelial growth factor (VEGF) (Buettmann et al., 2019; Hu & Olsen, 2017). The results of quantitative PCR in the bone tissue showed that the gene coding for VEGFA, but not those coding for VEGFB and VEGFD, was significantly downregulated in transcription of the Atg7^‐/‐^ mice (Figure 6a). Identification of H vessels by CD31 and EMCN markers revealed severe impairment in the hematopoietic autophagy‐defective mice (Figure 6b). Apparently, loss of H vessels is the upstream cause that leads to the disturbance of osteocyte homeostasis.

## 3 DISCUSSION

Previous studies on osteoporosis overwhelmingly focused on the etiology within bone tissue that locally induces the disease. In this study, we showed that osteoporosis is highly associated with reduction in hematopoietic autophagy activity in humans. With histomorphological, flow cytometric, functional, transcriptomic, and proteomic studies in a mouse model, we showed that an autophagy defect in the hematological system leads to severe bone loss. The disturbed osteocyte homeostasis is apparently caused by impaired‐type H vessels and possibly an aberrant alteration in the ECM pathways that govern osteocyte homeostasis in hematopoietic autophagy‐defective mice. Our results thus suggest that autophagy in the adjacent hematopoietic cells is essential to maintain bone homeostasis, and chronic hematopoietic autophagy deficiency can result in the development of osteoporosis in both mice and humans (Figure 6c).

While the osteal impact on hematopoiesis, in particular on the formation of bone marrow hematopoietic stem cell niches, is well documented (Mansour et al., 2012), studies of hematopoietic regulation of osteocyte function have been inadequate. Hematopoietic regulation of osteoblast proliferation and differentiation was previously discussed largely with skepticism speculation (Bethel, Srour, & Kacena, 2011). However, a recent study showed that loss of the hematopoietic stem cell factor GATA2 in the osteogenic lineage impairs trabecularization and mechanical strength of bone (Tolkachov et al., 2018). Our present study of RNA sequencing revealed that impaired HSPCs by autophagy defect also led to enhanced iron activity (Figure 5b), which may eventually lead to iron overload, a major cause of osteoporosis. In the induced osteoporotic mouse model generated by injection for iron overload, autophagy deterioration was observed in hematopoietic cells (Figure 2e), supporting the results identified in human osteoporotic patients (Figure 1).

Bone loss in hematopoietic autophagy‐defective mice does not result from an embryonic bone developmental defect, because genetic disruption of hematopoietic autophagy did not impair the skeletal system in postnatal mice (Figure 3i); rather, it accelerates bone aging, as manifested by aging hallmarks such as accumulated ROS and increased DNA damage (Figure 4) (Lean et al., 2003; Manolagas, 2010; Onal et al., 2013; Shi, Wu, Yan, Wang, & Miao, 2015; Yang et al., 2014). This suggests that the impairment of bone homeostasis by hematopoietic dysfunction in autophagy occurs along with accelerated aging in mice. This observation is consistent with the fact that osteoporosis develops primarily in the aged human population (Reginster & Burlet, 2006).

Hematopoietic cells and osteocytes are adjacent in the bone marrow niche environment. Normal hematopoiesis and bone homeostasis are interdependent (Alvarez et al., 2018; Divieti Pajevic & Krause, 2019; Vi et al., 2015). Men with low BMD or greater BMD loss have decreased circulating erythrocytes and lymphocytes and increased myeloid cells, and anemia or low blood cell counts are associated with declining BMD or increased fracture risk in the aged population (Valderrabano et al., 2017). Bone loss was also observed in an acute myeloid leukemia animal model, and osteogenesis was inhibited in leukemic mice (Frisch et al., 2012). On the other hand, chronic disorders affecting hematopoiesis, such as sickle cell anemia and thalassemia, demonstrate clear skeletal phenotypes, including bone loss and increased fracture risk (Valderrabano & Wu, 2019).

Previous studies show that transplantation of HSCs into lethally irradiated mice reconstitutes the defective osteoprogenitor pools. In particular, bone marrow HSCs were found to be capable of differentiating to osteoblasts through a mesenchymal intermediate (Olmsted‐Davis et al., 2003). These findings suggest that the developmental capacity of HSCs is not restricted to hematopoietic lineages, but extends to osteogenic differentiation, possibly via the HSC potential to trans‐differentiate to osteocytes. HSCs do not rest passively in their niche, but instead directly participate in bone formation and niche activities (Jung et al., 2008). Therefore, HSC functions and bone turnover are coupled in osteoporosis. Osteoblasts can also be regulated by HSC intercellular transfer mechanisms that rely on signaling endosomes, in which the HSC membrane surface antigens CD133 and CD63 are phagocytosed by osteoblasts, leading to downregulation of Smad signaling and upregulation of the chemokine ligand CXCL12 (Gillette, Larochelle, Dunbar, & Lippincott‐Schwartz, 2009). In the present study, we did not determine whether aggravation of osteoporosis is a direct consequence from autophagy deficiency in HSCs or other hematopoietic cells and still needs further study.

Major cellular functions of autophagy aid in its control of oxidative stress by limiting mitochondrial mass through autophagic degradation. A disruption or a deficiency in autophagy causes old mitochondria to accumulate. This directly leads to an elevated level of ROS (Lean et al., 2003; Manolagas, 2010; Shi et al., 2015; Yang et al., 2014). Long‐lasting accumulated ROS is a key upstream factor that triggers osteoporosis (Ezzat et al., 2018; Ma et al., 2018). Although autophagy remained normal in bone tissue, mitochondrial mass and ROS levels increased in both bone tissue and hematopoietic tissue of the mice with disrupted autophagy in their hematopoietic system (Figure 3 and Figure S8), suggesting that an alternative mechanism may exist in the functional connection between the hematopoietic system and bone homeostasis.

H vessels are significantly decreased in aged mice, and bone loss in the aged mice can be significantly reversed with drugs for angiogenesis, and therefore, angiogenesis and osteogenesis are coupled by H vessels (Kusumbe, Ramasamy, & Adams, 2014). In OVX‐induced osteoporotic mice, H vessels are also decreased (Xie et al., 2014). However, pharmacological increase in H vessels by harmine treatment prevents bone loss in the OVX mice (Huang et al., 2018). Consistent with the above findings in mice, our previous study showed a reduction of H vessels in the skeleton of an aged human population, and the type H vessels can be used as a sensitive biomarker for human bone mass (Wang et al., 2017). Apparently, type H vessels serve a critical functional connection between the hematopoietic system and bone tissue. In this study, we found that an autophagy defect in the hematopoietic system resulted in a severe loss of the vessels in the bone tissue. Therefore, loss of H vessels due to hematopoietic autophagy insufficiency is very likely the direct upstream defect that leads to disrupted bone homeostasis, and ultimately osteoporosis.

This conclusion may be strengthened by comparing the restorative effect by pharmacological treatment with autophagy inducers between hematopoietic autophagy gene Atg7 knockout mice and osteoporotic mouse model generated by OVX or FAC injection. However, it is currently a challenge to specifically activate in vivo hematopoietic autophagy with autophagy inducers commercially available. Therefore, administration of pan autophagy inducers, such as rapamycin, is expected to activate all types of Atg7 wild‐type cells including osteocytes in the hematopoietic Atg7‐deleted mice. This will ultimately lead to a reduced osteoporosis in the Atg7‐deleted mice, weakening the reliability of the Atg7‐deleted mice for use as a negative control for hematopoietic‐specific rescue effect in the osteoporotic model mice. Nevertheless, our recent work shows that activation of autophagy by injection of rapamycin recovered angiogenesis and osteogenesis in the induced osteoporosis mouse model, which was generated by injection of ferric ammonium citrate for iron overload (Wu et al., 2019). This finding generally supports our present contention that loss of autophagy in hematopoietic system compromises angiogenesis and osteogenesis.

Overall, our finding that osteocyte disorder is caused by the disruption of H‐type vessels from a defective capacity for hematopoietic autophagy adds new evidence that links the hematopoietic system to bone homeostasis that depends on hematopoietic autophagy, but search on specific cell type responsible for the hematopoietic connection to osteoporotic phenotype yet remains an open scenario for our future work. Nevertheless, given the interconnection between hematopoietic autophagy and bone homeostasis identified here, it is therefore important to examine and improve hematopoietic autophagy function when considering clinical therapeutic interventions for osteoporosis.

## 4 EXPERIMENTARY PROCEDURES

### 4.1 Clinical research

A total of 4,979 individual physical examination results were analysis for blood count between osteoporosis and nonosteoporosis. The physical examination information is from Physical Examination Center, the Second Affiliated Hospital of Soochow University. Osteoporosis was defined based on bone mineral density (BMD), per WHO standards (BMD *T* value <−2.5). Areal BMD in hip and spine was measured by dual‐energy X‐ray absorptiometry (DXA), and the RBC counts were obtained from routine blood tests.

Human femur bone marrow samples were from patients with surgery between 48 and 87 years old (age 70.0 ± 10.5). Bone marrow was collected during hip replacement surgery or knee replacement surgery. Patients who had hip fractures caused by trauma or failing, and osteoarthritis were included in our study (inclusive criteria). Subjects with secondary osteoporosis, anti‐osteoporosis drug treatment, a disrupted hematopoietic system, malignancy, diabetes, or other severe diseases in the previous 5 years were excluded from our study (exclusive criteria). All clinical procedures were approved by the Ethics Committees of the Second Affiliated Hospital of Soochow University. We also obtained informed consent from the participants. Bone marrow was immediately stored in a 4°C freezer until it was used for analysis. BMD was tested by dual‐energy X‐ray absorptiometry. Blood cells were counted using SPSS 25.0. Monocytes were collected by Ficoll and stained for CD34 (BD) and CD45 (BD). Cell staining was performed first with 594 conjugated LC3 and LAMP‐1 (Abcam), followed with Alexa Fluor 649 goat anti‐rabbit antibody (Multi Sciences) and then analyzed with an ImageStreamX MarkII (Amnis).

### 4.2 Animal studies

Mice lacking autophagy in the hematopoietic system were generated by crossing mice hemizygous for a Vav‐Cre transgene (Jackson Laboratory) with mice heterozygous for an Atg7‐flox allele provided by Dr. Komatsu (Komatsu et al., 2005) to generate heterozygous Atg7‐flox offspring with and without the Vav‐Cre allele. These offspring were crossed to generate wild‐type mice (Atg7^+/+^), mice hemizygous for Vav‐Cre allele, and mice homozygous for the Atg7‐flox allele, with Atg7 deletion in the hematopoietic system (Atg7^−/−^) (Cao, Zhang, Cai, et al., 2015; Cao, Zhang, Yuan, et al., 2015; Mortensen et al., 2011). Genotype was verified by PCR of tail DNA samples. For the ovariectomy (OVX) model, 8‐week‐old C57 female mice were bilaterally ovariectomized and sacrificed at 14 weeks old. For the iron overload model, 8‐week‐old C57 male mice were intraperitoneally injected with 0.1 g kg^−1^ week^−1^ of ferric ammonium citrate (FAC) for 8 weeks. The mice were bred and housed in the specific‐pathogen‐free animal facilities of Soochow University. All animal experiments were approved by the institutional animal care and use committee of Soochow University.

### 4.3 Western blotting

Bone marrow cells were collected from femur and tibia, and cell extracts were prepared for Western blotting as previously described (Cao, Zhang, Yuan, et al., 2015).

### 4.4 Skeletal analysis

Bone mineral density (BMD) and trabecular spatial structure parameters were analyzed using a micro‐CT scan (SkyScan 1176). Femora were dissected after the soft tissue was removed and then stored in 4% paraformaldehyde. The scanners were set at a voltage of 50 kV, a currency of 500 μA, and a resolution of 9 μm. Image software (NRecon v1.6) and data analysis software (CTAn v1.13.11.0 and 1.11.10.0) were used for analysis of BMD and for 3D reconstruction of the trabecular bone (Yuan et al., 2019). The constant region of interest (ROI) was defined, and 3D images of trabecular bone were reconstructed based on the ROI. The outlined ROI was selected from 540 μm proximal to the distal growth plate and extended 1.35 mm toward the diaphysis. BMD was calculated within the ROI. Parameter analysis included BMD, trabecular number (TB.N), bone tissue fraction (BV/TV), trabecular thickness (Tb.Th), cortical thickness, and trabecular space (Tb.Sp).

### 4.5 Biomechanical properties

Biomechanical properties of femora were measured using three‐point bending and compression testing. Femora were collected and stored at −20°C. Femora were tested in three‐point bending with the posterior surface on the lower supports (5 mm apart) and the load applied to the anterior surface centered between the lower supports, as previously described in detail (Akhter, Cullen, Gong, & Recker, 2001). Biomechanical structural strength variables were measured, including ultimate load, stress, stiffness, and strain.

### 4.6 Bone histology and immunohistochemistry

Tissues were fixed in 10% formalin overnight, decalcified by 20% EDTA for 2 weeks, and processed for paraffin sectioning. Tibiae were collected and fixed overnight at 4°C and embedded undecalcified in methyl methacrylate. Sections 5 μm thick were processed for hematoxylin and eosin (H&E) staining. For immunohistochemistry (IHC), sections were incubated in citrate buffer (10 mM citric acid, pH 6.0) overnight at 60°C to unmask antigens, then incubated with primary antibodies Collagen 1 (Servicebio) and Masson kit (Ccell) overnight at 4°C and secondary antibodies for 1 hr at room temperature.

### 4.7 Calcein labeling

Mice were administered by intraperitoneal injections of calcein at a dose of 30 mg/kg in a 1% saline solution, respectively, for 7 and 2 days before they were sacrificed. Femur was fixed overnight in 4% paraformaldehyde overnight, dehydrated in 30% sucrose for 2 days, and sectioned for calcein (Everbright Inc) labeling (Vi et al., 2015).

### 4.8 Skeleton staining

Mouse skin and organs were removed and fixed for 4 days in 95% ethanol and then in 2% KOH for transparency. The skeleton was stained with Alcian blue solution (Sinopharm Chemical Reagent Co., Ltd; 10 mg Alcian blue 8NG + 80 ml 95% ethanol + 20 ml acetic acid) and Alizarin red S (Sinopharm Chemical Reagent Co., Ltd) (10 ml Alizarin red S saturated ethanol solution with 0.9 g KOH in 90 ml water solution). Skeletons were transferred to glycerol and imaged.

### 4.9 Morphology analysis by scanning electron microscopy

Femora were used for scanning electron microscopy (SEM). Tissues were fixed in 2.5% glutaraldehyde in PBS for 24 hr at 4°C. They were dehydrated in a series of ethanol solutions. Samples were fixed on stubs with carbon tape and sputter‐coated with gold in an ion coater. The morphology of the granules was examined under vacuum with an SEM.

### 4.10 ROS level detection

Bone tissue was digested with trypsin and collagenase I to collect cells. Cells were washed in FBS before loading with DCFH‐DA (Sigma) in PBS. After two washes in PBS, cells were incubated for designated times in the figures with ACR prior to measurement by flow cytometry. Logarithmic amplification was used to measure DCFH‐DA fluorescence.

### 4.11 Bone immunostaining

Tibiae were fixed in 4% paraformaldehyde overnight at 4°C. The specimens were used for decalcification by EDTA solution for 4 weeks and then dehydrated using 20% sucrose and 2% polyvinylpyrrolidone solution for 2 weeks. The specimens were embedded in O.C.T. tissue freezing medium (Leica) for 10 μm frozen sectioning. Thick frozen sections were rinsed by PBS with 0.1% Triton three times before staining. Sections were incubated with primary antibodies Alexa Fluor 488‐phalloidin (Molecular Probes), MitoTracker Deep Red (Invitrogen), H2AX (Cell Biolabs, Inc), and ATG7 (Cell Signaling Technology) at 4°C overnight. After three washes with PBS containing 0.1% Triton, the ATG7 sections were incubated with fluorescein‐conjugated secondary antibodies and other sections together with nuclear counterstaining dye (DAPI; Beyotime) at room temperature. After three more washes, slides were covered, and images of immunostaining were acquired using a confocal microscope.

### 4.12 RT–qPCR analysis

Human bone marrows were collected in surgery detailed in the clinical research section. Mouse tibia was dissected after removing soft tissue. Total RNA was prepared from bone tissue by liquid nitrogen or human cells and mixed in 1 ml TRIzol. cDNA was reverse‐transcribed using a reverse transcription kit. The following primers were used for human samples: Atg5, Atg7, Atg12, LC3b, Lamp2a, and P62. The following primers were used for mice samples: SP7, RUNX2, BMP2, BMP6, CTSK, TRAP5, VEGFA, VEGFB, and VEGFD. The primer–probe sets used are presented in Table S1. Relative mRNA expression levels were normalized by mouse GAPDH expression.

### 4.13 Flow cytometry for isolating hematopoietic cells

Bone marrow was isolated from femur and tibia. Flow sorting of hematopoietic cells and their stem and progenitor cells (HSC, HPC) were performed as previously described (Cao, Zhang, Yuan, et al., 2015). For enrichment of Lin^−^ cells, single‐cell suspensions of bone marrow cells were obtained by flushing femurs and passaging through PBS containing 2% FBS. Mononuclear cells were enriched by equilibrium centrifugation over a cushion of Ficoll‐Paque PLUS (GE Healthcare) by density gradient centrifugation. Lineage cells were harvested by magnetic‐activated cell sorting (MACS) according to the protocol of a mouse lineage depletion kit (Miltenyi Biotec), followed by sorting for HPCs and HSCs (LK, LSK, and LSKCD48^−^ CD150^+^ markers) with fluorescence‐activated cell sorting (FACS; Aria III, BD Biosciences). The subsequent HSCs or HPCs were analyzed with flow cytometry for cell apoptosis using a PI/Annexin‐V assay kit (BD Biosciences).

### 4.14 RNA sequencing analysis

LSK cells were sorted from 10‐week‐old Atg7^+/+^ and Atg7^−/−^ mice. Sequencing libraries were generated using NEBNext^®^ Ultra™ RNA Library Prep Kit for Illumina^®^ (NEB) following the manufacturer's recommendations and followed by sequencing on an Illumina HiSeq platform to generate 125 bp/150 bp paired‐end reads. Approximately 40 million paired‐end reads per sample were aligned to the mouse genome (GRCm38/mm10). STAR used the method of Maximal Mappable Prefix (MMP), which can generate a precise mapping result for junction reads. HTSeq v0.6.0 was used to count the read numbers mapped to each gene. Then, FPKM of each gene was calculated based on the length of the gene and read counts mapped to this gene. Differential expression analysis of two conditions/groups (two biological replicates per condition) was performed using the DESeq2 R package (1.10.1). DESeq2 provides statistical routines for determining differential expression in digital gene expression data using a model based on the negative binomial distribution. The resulting *p*‐values were adjusted using Benjamini and Hochberg's approach for controlling the false discovery rate. Genes with an adjusted *p*‐value < .05 and fold change >2 found by DESeq2 were assigned as differentially expressed. Gene Ontology (GO) enrichment analysis of differentially expressed genes (DEGs) was implemented by the cluster Profiler R package, in which gene length bias was corrected. GO terms with corrected *p*‐value < .05 were considered significantly enriched by DEGs. The RNA‐seq sequencing clean reads were deposited in GenBank database with an accession number PRJNA506815 (http://www.ncbi.nlm.nih.gov/bioproject/506815).

### 4.15 Proteomic profiling

With the data produced from the bone tissues of the wild‐type and autophagy‐defective mice, all targeted proteins and function‐related downregulated protein identifiers were searched against the STRING database version 10.5 for protein–protein interactions. Only interactions between the proteins belonging to the searched data set were selected, thereby excluding external candidates. STRING defined a metric called “confidence score” to define interaction confidence. We retained all interactions that had a confidence score ≥0.4 (medium confidence). The interaction network form STRING was visualized in Cytoscape. A graph theoretical clustering algorithm, molecular complex detection (MCODE) was utilized to analyze densely connected regions. MCODE is part of the plug‐in tool kit of the network analysis and visualization software Cytoscape.

### 4.16 Statistics

The clinical data of osteoporotic patients and healthy individuals were collected with blood cell count, age, and femur neck BMD. Pearson correlation coefficient was analyzed between femur neck BMD and age, RBC count and age, and femur neck BMD and RBC count, using SPSS 25.0. Experimental data were presented as means ± standard deviations (*SD*s), which was evaluated using Student's *t* tests.

## CONFLICTS OF INTEREST

The authors have declared no conflicts of interest.

## AUTHOR CONTRIBUTIONS

YY and YF designed the experiments and performed most of the experiments. LZ, YG, RZ, and LL did Western blotting, RT–qPCR, and flow cytometric analysis. YF and JQ did informatics analysis. PZ and JL collected human samples. YY and HZ generated the OVX mouse model. NY, SZ, and QM prepared the knockout mice and mouse specimens. YY, YF, JW, and YX analyzed the data and wrote the manuscript. JW and YX supervised the study.
