# Supplementary material for: Deterioration of hematopoietic autophagy is linked to osteoporosis
Source: Aging Cell. 2020 Mar 25;19(5):e13114. doi: 10.1111/acel.13114 (PMC7253060; doi:10.1111/acel.13114)
Supplement: Supplementary file 1 [file ACEL-19-e13114-s001.pdf]

Supplementary Information

Deterioration of hematopoietic autophagy is linked to osteoporosis

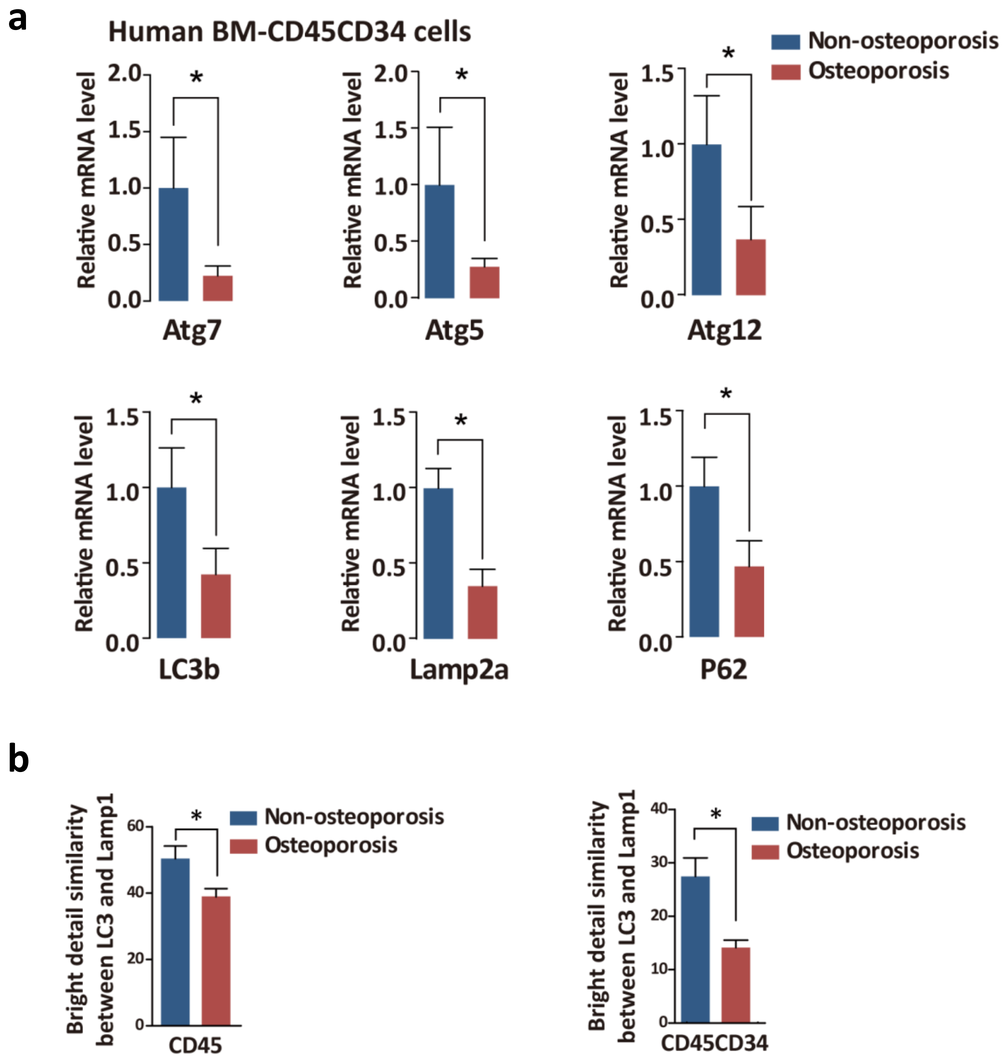

Figure S1: Bone marrow hematopoietic autophagy is decreased in female osteoporotic patients. (**a**) Expression of autophagy-essential genes in the bone marrow hematopoietic and stem cells of female osteoporotic patients and healthy individuals. Human femoral bone marrows were collected in total hip replacement or total knee replacement surgery. Bone marrow hematopoietic stem and progenitor cells were isolated with magnetic-activated cell sorting against CD45 and CD34 antibodies after Ficoll-gradient separation. mRNA levels of Atg7, Atg5, Atg12, LC3b, Lamp2a and P62 genes were detected by real-time quantitative PCR. BMD T value < -2.5 is a criterion for osteoporosis. (**b**) Detection of autolysosome formation in healthy and osteoporotic population. Autolysosome formation was measured by image flow cytometry for double staining of LC3 and lysosome with bone marrow CD45 or CD45CD34 cells prepared by fluorescence-activated cell sorting. Autolysosome formation was represented by the colocalization of LC3 and lysosomal marker LAMP1. Date = means ± SDs. *p<0.05.

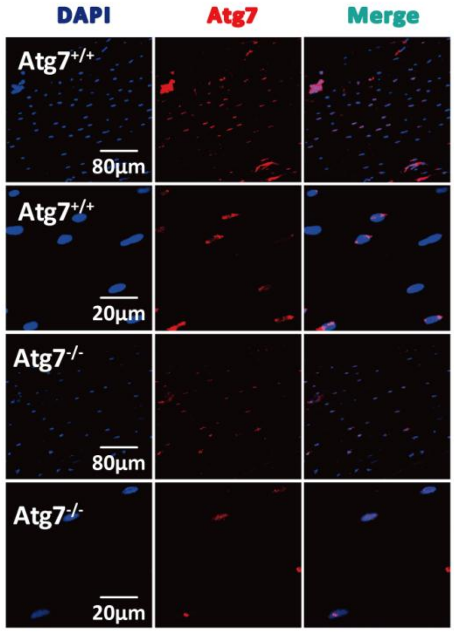

Figure S2. Immunofluorescence of mouse tibia cortical bone stained with ATG7 antibody. Tibia were collected from 8-week-old mice for frozen section and immunohistochemistry. Fluorescently-tagged ATG7 staining (red) was performed to detect the expression of ATG7 in the mouse tibia. Nuclei were visualized by DAPI staining.

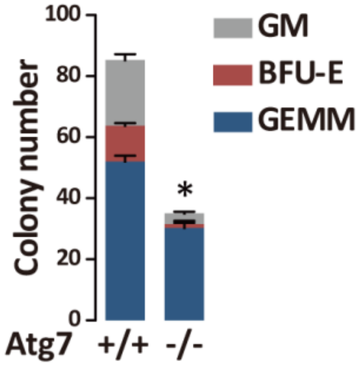

Figure S3. In vitro proliferation and self-renewal analysis on the mouse hematopoietic stem and progenitor cells by CFU assay. Bone marrow HSPCs (sorted against LSK markers) from 8-week-old mice were isolated from the mice indicated for CFU assay .

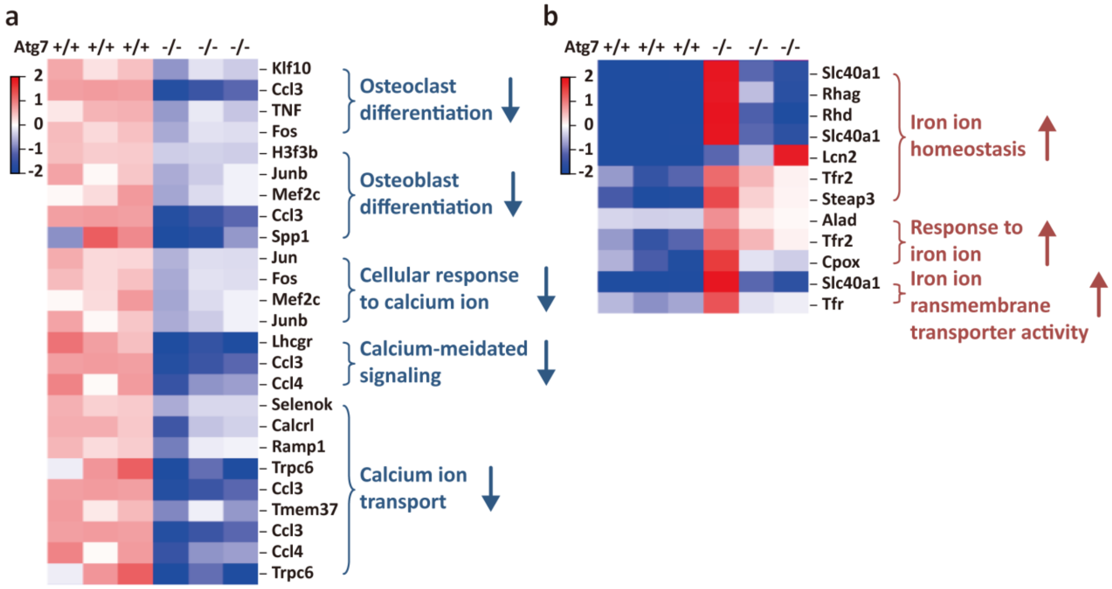

Figure S4. Transcriptional analysis of mouse HSPCs on calcium metabolism and osteocyte differentiation as well as iron response. Shown are heatmaps of gene expression levels for osteoclast and osteoblast differentiation and calcium metabolism (**a**) and iron homeostasis (**b**) in the HSPCs (sorted with LSK markers) by RNA-sequencing. The HSPCs were sorted from 10-week-old Atg7$^{+/+}$ and Atg7$^{-/-}$ mice. The RNA sequencing protocol is described in detail in the methods.

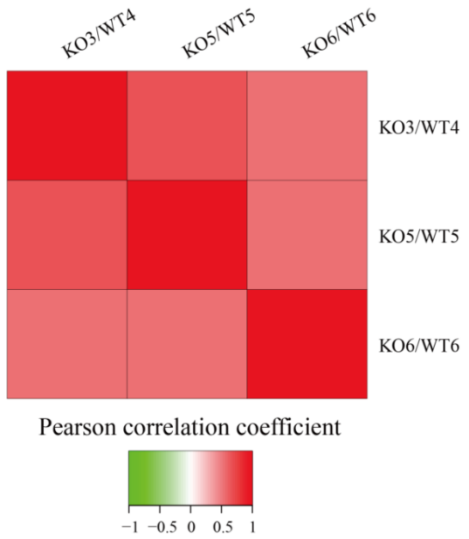

Figure S5. Repeatability checked in each sample. Pearson correlation coefficient is calculated between each sample. The closer to 1, the more positive the correlation is.

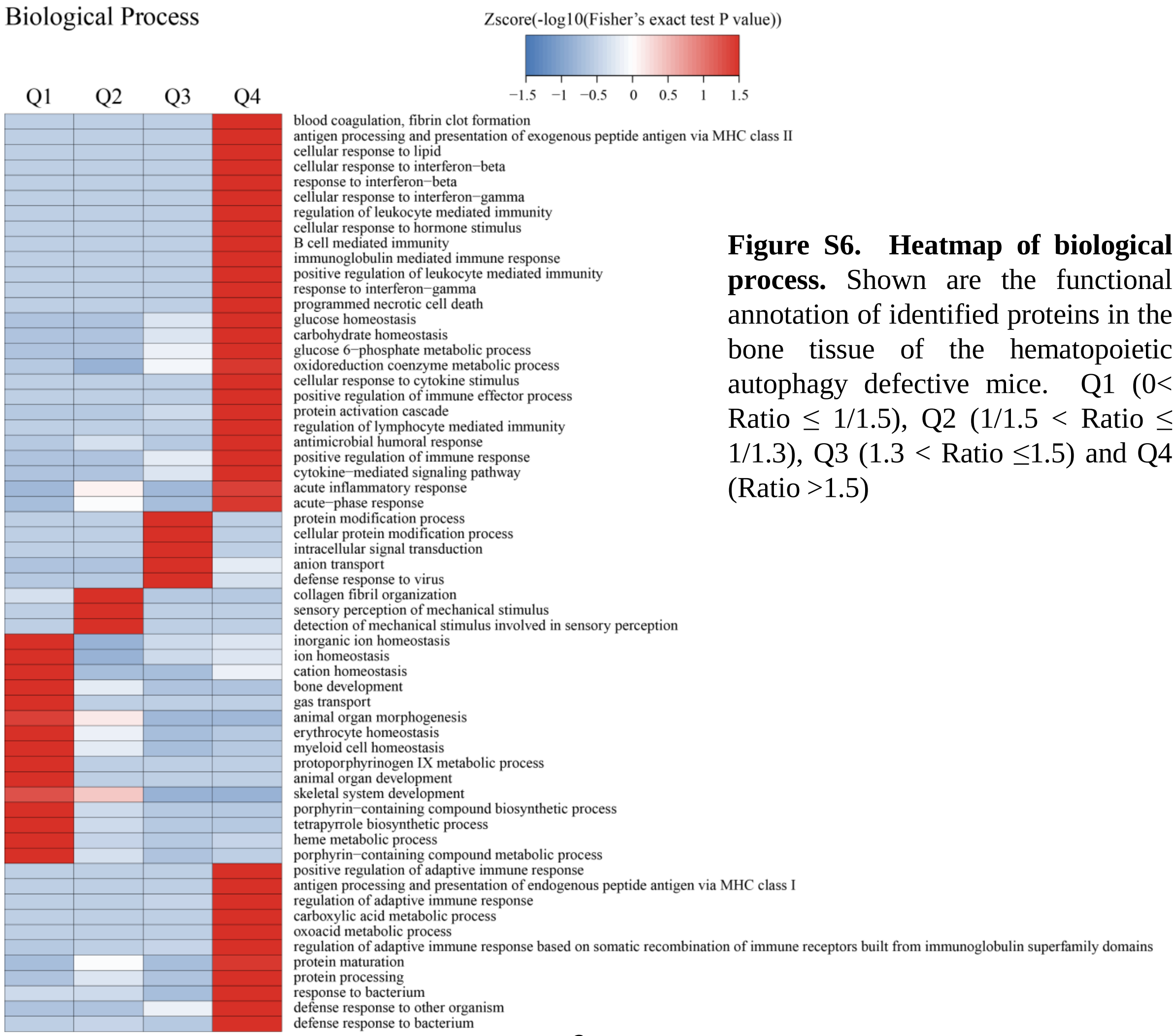

Figure S6. Heatmap of biological process. Shown are the functional annotation of identified proteins in the bone tissue of the hematopoietic autophagy defective mice. Q1 ($0 <$ Ratio $\leq 1/1.5$), Q2 ($1/1.5 <$ Ratio $\leq 1/1.3$), Q3 ($1.3 <$ Ratio ≤ 1.5) and Q4 (Ratio > 1.5)

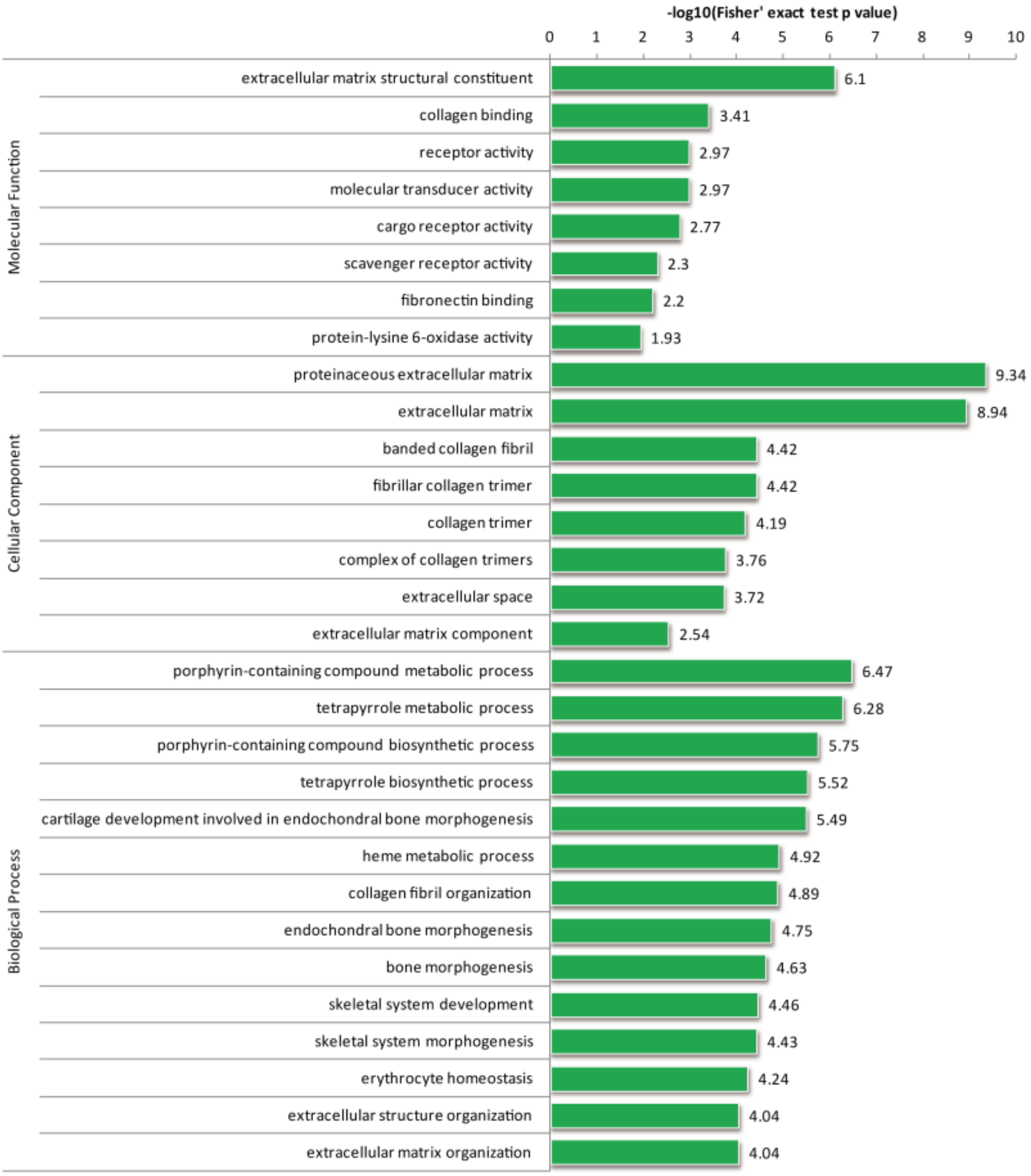

Figure S7. Osteogenesis protein inhibited in Atg7$^{-/-}$ mice bone tissue. Functional annotation of identified down-regulated proteins in Atg7$^{-/-}$ mice bone tissue were displayed. Selected GO terms for cellular components, molecular functions and biological processes were displayed. n=3.

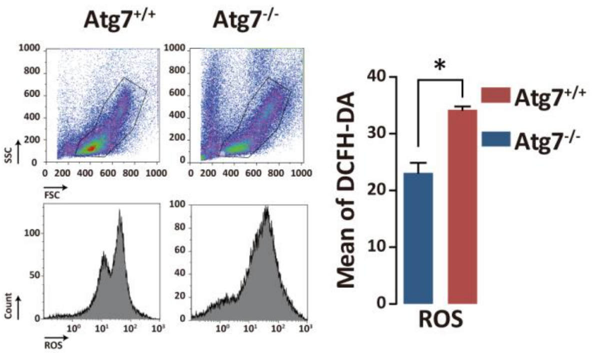

Figure S8. Flow cytometric measurement of ROS levels in total bone marrow hematopoietic cells. Bone marrow total hematopoietic cells from 8-week-old mice were collected for cytometric detection of ROS level. Logarithmic amplification was used to measure DCFH-DA fluorescence.

Table S1. Primers used for RT-PCR

Gene	Primers (forward/ reverse)
hAtg7	(F) 5'-TAATGTCCTTCCCGTCAGCCT-3'
	(R) 5'-TCATGTCCCAGATCTCAGCAG-3'
hAtg5	(F) 5'-CAACTTGTTTCACGCTATATCAGG-3'
	(R) 5'-CACTTTGTCAGTTACCAA CGTCA-3'
hAtg12	(F) 5'-AAGTGGGCAGTAGAGCGAAC-3'
	(R) 5'-CCATCACTGCCAAAACACTCA-3'
hLC3b	(F) 5'-TGTCCGACTTATTCGAGAGCAGCA-3'
	(R) 5'-TTCACCAACAGGAAGAAGGCCTGA-3'
hLamp2a	(F) 5'-GCACAGTGAGCACAAATGAGT-3'
	(R) 5'-CAGTGGTGTGTATGGTGGGT-3'
hP62	(F) 5'-GACTACGACTTGTGTAGCGTC-3'
	(R) 5'-AGTGTCCGTGTTTCACCTTCC-3'
Bmp2	(F) 5'-CCCACTTGGAGGAGAAACAA-3'
	(R) 5'-AGCCACAATCCAGTCATTCC-3'
Bmp6	(F) 5'-AAGGCTGGCTGGAATTTGACATCACG-3'
	(R) 5'-GGTAGAGCGATTACGACTCTGTTGTC-3'
Sp7	(F) 5'-AGGAGGCACAAAGAAGCCATAC-3'
	(R) 5'-GATGCCTGCCTTGTACCACGAGC-3'
Runx2	(F) 5'-AACTTCCTGTGCTCCGTGCTG-3'
	(R) 5'-TCGTTGAACCTGGCTACTTGG-3'
Ctsk	(F) 5'-TGTATAACGCCACGGCAAA-3'
	(R) 5'-GGTTCACATTATCACGGTCACA-3'
Trap5	(F) 5'-TACCTGTGTGGACATGACC-3'
	(R) 5'-CAGATCCATAGTGAAACCGC-3'
Vegfa	(F) 5'-CAAGATCCGCAGACGTGTAA-3'
	(R) 5'-CTGTCAACGGTGACGATGAT-3'
Vegfb	(F) 5'-GGTGCCATGGATAGACGTT-3'
	(R) 5'-AGGATCTGCATTCGGACTTG-3'
Vegfd	(F) 5'-AGCATGTCAGGGCCATTT-3'
	(R) 5'-GAGCTGTTTGGAGATGTAGGAG-3'
