# Supplementary figures and images for: Deterioration of hematopoietic autophagy is linked to osteoporosis
Source: Aging Cell. 2020 Mar 25;19(5):e13114. doi: 10.1111/acel.13114 (PMC7253060; doi:10.1111/acel.13114)

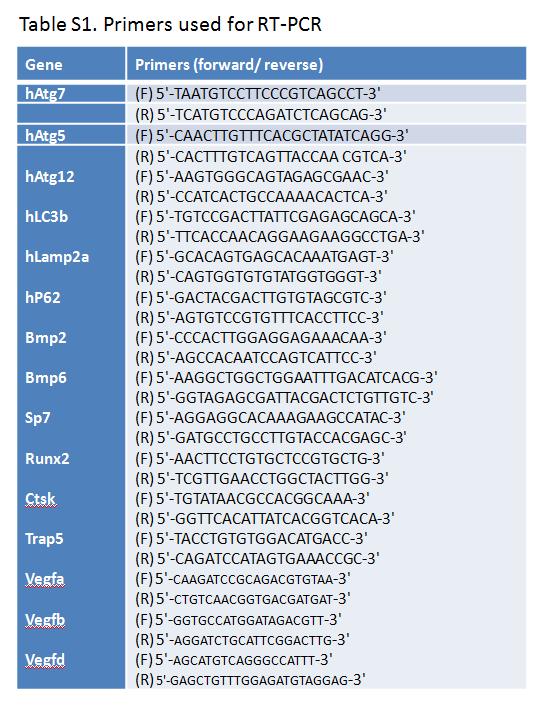

Supplement: Supplementary file 2 [file ACEL-19-e13114-s002.jpg]
